# Differential Survival among Batches of Atlantic Cod (*Gadus morhua* L.) from Fertilisation through to Post-Metamorphosis

**DOI:** 10.1371/journal.pone.0158091

**Published:** 2016-06-30

**Authors:** Petra E. Petersen, David J. Penman, Geir Dahle, Øystein Patursson, John B. Taggart

**Affiliations:** 1 Department of Biotechnology, Aquaculture Research Station of the Faroes, við Áir, Hvalvík, Faroe Islands; 2 Institute of Aquaculture, School of Natural Sciences, University of Stirling, Stirling, Scotland; 3 Institute of Marine Research, Nordnes, Bergen, Norway; Ecole normale superieure de Lyon, FRANCE

## Abstract

Aquaculture production of cod has decreased from over 20,000 tonnes in 2009 to less than 2,000 tonnes in 2014 and the industry faces many challenges, one of which is high and unpredictably variable mortality rates in the early life stages. Hence, full-cycle farming with hatchery produced juveniles is still considered unprofitable compared to fisheries and on-growing of wild cod. In the present study, potential batch differences in progeny survival of wild-caught, hatchery-spawned Faroe Bank cod (*Gadus morhua* L.) were investigated at two defined periods during early life history; i) the embryo stage (60 day degrees post fertilisation) and ii) the fry stage (110 days post hatch), post metamorphosis. The fry stage experiment was conducted in three replicates (*N* = 300 per replicate), and a panel of three polymorphic microsatellite markers was used for parental analysis. Mean survival rate at the embryo stage was 69% (± 20% SD). Survival was positively associated with egg diameter (*P* < 0.01), explaining 90% of the variation in egg survival rates. The data were too scarce to conclude either way concerning a possible correlation between survival rates between the two periods (*P* < 0.10). Offspring from three batches (from a total of eight) dominated in the fry stage, contributing over 90% of the progeny, and results were consistent over all three replicate tanks. The skewed batch representation observed may be of relevance to the effective management of selective breeding programmes for cod.

## Introduction

The Atlantic cod has the potential to become and important aquaculture species, though the production of cultured cod has not reached the levels anticipated a decade ago. One reason is the constant competition with the wild cod fisheries and vulnerability to fluctuations in the world economics and fish prices. A decade ago, production of cultured cod was growing in Canada, USA, Iceland, the Faroe Islands and Norway. Following the recent global financial crisis, however, production of cultured cod decreased from over 20,000 tonnes in 2009 to less than 2,000 tonnes in 2014, mainly produced by Norway [1,2]. The industry is also faced with many biological and technical challenges, including a variety of infections and diseases, early sexual maturation accompanied by growth loss, escape of fish and eggs from sea cages as well as the need for more cost-efficient feeds produced from sustainable sources [3,4]. Furthermore, intensive production of Atlantic cod still involves high and unpredictably variable mortality rates in the early life stages [5], particularly from first-feeding through to metamorphosis, where there is a lack of knowledge on optimal rearing protocols [6]. Hence, although closed life cycles have been established for Atlantic cod [4,7], full-cycle farming with hatchery produced juveniles is still considered unprofitable compared to fisheries, on-growing of wild cod in sea cages and ranching and feeding wild cod in herds [8].

The cod mating system can be classified as a lekking system [9,10], prerequisites of which include male / male competition and non-random mating by females [9]. In addition to sexual traits, characteristics typically considered important to successful mate choice include female and male body size and condition [9,11,12,13,14] as well as male / female size differences [13,15,16,17]. Irrespective of the precise mechanisms involved in determining mate choice, the outcome appears to be a skewed parental contribution to the progeny generation, as seen in both commercial [18,19] and experimental setups (for example [9,20]). As individual female cod can release up to around 20 egg batches over one to two months [21], only a proportion of the females can be expected to contribute to each spawning bout. For example, an investigation of paternity in 300 fry at 83 days post hatch (dph), originating from eggs collected on a single day from a commercial breeding tank containing 99 parental cod, found that only 26 parental cod had contributed to the assigned progeny. Furthermore, 81% of the progeny were assigned to a single pair of parents [18]. This family skew in reproduction can also be seen on a seasonal basis. In a study monitoring egg batches generated in a mass spawning tank containing 15 female and 15 male cod over an entire breeding season, it was reported that just five females and five males contributed to 77 and 54% of the sampled eggs, respectively [22]. Lastly, from an experimental setup of six enclosures, each containing an average of four females and four males, a total of 1340 progeny from 102 spawning events distributed across the breeding season were analysed. This study discovered a shift in male reproductive dominance, likely explained by sperm depletion in high-ranking males, thereby allowing second ranking males to take over. However, the seasonal average reproductive success for males still varied from close to zero progeny sired by some males to nearly 90% progeny sired by one of the males [13].

There is a need to monitor and understand the factors underlying the variability in survival among egg batches in order to obtain a stable supply of juveniles for the aquaculture industry. Egg morphology, biochemical composition and early cleavage patterns amongst others may be useful indicators of egg quality, as seen from a number of studies on cod and other species [23,24,25,26]. In a study of Atlantic cod eggs [27], six batches were classified into normal and abnormal cleavage patterns and significantly higher cumulative egg mortalities were found for the abnormal eggs. However, abnormal and normal eggs did not differ in hatching success (proportion eggs that hatched of those that survived up to the weighted mean time to hatch), larval deformity rates and larval mortality rates within 24 hours of hatch.

The present study was undertaken in order to investigate whether survival rates of cod batches at the embryo stage could be correlated to survival rates in metamorphosed fry and to investigate potential predictors (egg size and female condition) of survival rates in these two stages. The experimental setup involved mating wild caught Faroe Bank cod in a set of eight tanks, each containing a single female and one or two males. Spawning batches were collected and survival rates estimated at two timepoints in development, 1) 60 day degrees post fertilisation, when survival was likely to be representative of hatching survival, and 2) at 110 dph, when all fry had undergone metamorphosis, three replicate tanks were applied in the fry stage.

## Materials and Methods

### Ethics statement

All procedures followed normal aquaculture practice except taking fin biopsy samples from spawners and killing fish by anaesthetic overdose. These procedures were undertaken under the supervision of the fish veterinarian licensed by the Aquaculture Research Station to supervise fish welfare. One of the authors, G. Dahle, holds an official license for animal experimentation (Category C from FELASA, the Federation of European Laboratory Animal Science Associations).

The Marine Research Centre of the Aquaculture Research Station is licensed by the Faroe Islands Food- and Veterinary Agency (FFVA) to perform experimentation with marine fish. FFVA manages the food and veterinary disease laws for the Ministry of Foreign Affairs and Trade. The specific study protocol was not reviewed prior to the start of the study, as no specific permits or ethical approvals were required for this study. Atlantic cod is listed as “vulnerable” on the IUCN Red List of threatened species. The parental fish used in this study were part of the Aquaculture Research Station broodstock, which were obtained as part of a Faroese Government licensed effort quota for small longliners and jiggers.

### Experimental design

Rearing protocols were modified from existing industry practices [28,29]. Faroe Bank broodfish were collected from April through August 2008 by local fishers using jigging reels. The fish were transported to the Marine Research Centre of the Aquaculture Research Station of the Faroes, where they were kept in outdoor flow-through tanks and allowed to adapt to captivity. In January 2009 the fish were PIT tagged, sex determined by ultrasound, weighed and measured (total body length) and fin clips were taken and stored in 96% ethanol at 4°C for DNA analysis. Prior to any handling, the broodfish were anaesthetised to “handleable” (defined as a loss of equilibrium and loss of reactivity to external stimuli) with a 100 mg l^-1^ of the anaesthetic agent metacaine (MS-222). The fish were only handled for the minimum amount of time necessary and immediately after treatment were put in running seawater to recover. On 31 March 2009 the fish were introduced into indoor tanks, maintained at ambient photoperiod, temperature and salinity (35 ‰[30]). The tanks were of circular, light grey, fibreglass reinforced plastic construction, 1.3 m diameter × 1.5 m high and contained approximately 1.5 m^3^ seawater, which was filtered (100 μm) and pumped into the tanks at a mean flow rate of 7 l min^-1^. Egg collectors fitted with a 500 μm mesh net, which retained all eggs, were attachted to the effluent water of each tank. To minimise the occurrence of failed spawning units due to possible male infertility and to stimulate more regular female spawning events, spawning units comprised one female and two males, with two exceptions (Table 1). Thus, full- and half-sib families could potentially be produced in most tanks. Parents were size matched as best as possible from available broodstock, based on the knowledge that matings with males of similar or slightly larger body lengths than the females are generally the most successful [13,18]. The fish were let to spawn spontaneously and were not fed while in the spawning tanks. Water temperature was relatively constant during spawning with an average of 7.0°C (± 0.3 SD).

**Table 1:** Mating design and sizes of pre-spawning parents.

ID	Length (cm)	Weight (kg)	*K*
F1	87.5*	10.3	1.54
M1a	84.0*	9.3	1.57
M1b	76.0	5.5	1.25
F2	80.5	8.9	1.71
M2a	82.0	8.6	1.56
M2b	83.0	8.5	1.49
F3	77.5	10.2	2.19
M3a	79.0	6.5	1.32
M3b	81.0	7.0	1.32
F4	87.0	7.5	1.14
M4a	85.0	8.9	1.45
M4b	85.0	9.0	1.46
F5	91.5	10.6	1.38
M5a	93.0	14.4	1.79
M5b	94.0	12.5	1.50
F6	97.0	12.7	1.39
M6a	94.5	12.9	1.53
M6b	95.0	11.0	1.28
F7	81.5	7.2	1.33
M7	83.0	8.9	1.56
F8	100.5	16.0	1.58
M8	94.5	11.4	1.35
Female averages	88 (± 8 SD)	10.4 (± 2.9 SD)	1.53 (± 0.32)

F = female; M = male; *K* = Fulton´s condition factor calculated as 100,000 × body mass (g) divided by [total length (mm)]^3^.

*These data were obtained later than the rest, on the day that the fish were introduced into the spawning tanks.

#### The embryo stage

In order to prevent cannibalism among the larvae, egg batches were collected on two consequtive days, 17 and 18 April. One single batch was obtained per spawning unit, i.e. a total of eight batches. Per batch, two deciliters of live (floating) eggs were transferred to incubation and batches were kept separately throughout incubation. Upon collection, batch-average egg diameters of a random sample of 50 eggs were assessed by stereomicroscopy and eggs per volume calculated (where numbers of eggs per ml = 1,222 × *D*^-2.71^, *D* = egg diameter (mm) [11]). Furthermore, stereomicroscopy assessment showed that the eggs were fertilised and that time of day, that spawning occurred, varied among the batches. On both days, eggs were collected between 13.30 and 15.00 and multiple cell divisions (> 32 cells) were observed for two batches, one batch was in the two cell stage, three in the four cell stage, one in the eight cell stage, while a single batch had not undergone any cell divisions yet. Prior to, and at the end of, incubation the eggs were surface disinfected with glutaraldehyde at 400 ppm for 8 min [31]. Incubation was achieved in black 15 l cylinders with a cone-shaped bottom, containing filtered (100 μm) and UV-treated seawater. The incubation temperature was kept constant at 5°C, central air stones provided constant aeration and circulation and one third of the water was exchanged daily. Average light intensity at the surface of the incubation cylinders was 210 lux (± 60 SD). Dead eggs became white and sunk to the bottom where they were siphoned out from the cylinders daily.

Egg volumes were used to apportion equal numbers of embryos from each of the eight batches in communal first-feeding tanks, and this factor largely dictated the first sampling time point, which had to be achieved prior to hatch. Hatching data from previous studies at the Marine Research Centre indicated that Faroe Bank larvae hatch after approximately 14 days at 5°C; and therefore on 29 April (60 day degrees post fertilisation), *c*. 45,400 eggs, comprising a similar number from each of the eight females (*c*. 5,675), were introduced into each of three first-feeding tanks. At this point, the percentage of embryos surviving incubation was estimated as percentage volume surviving of the initial 2 deciliter eggs transferred to incubation. The first-feeding tanks were black on the inside, constructed from fibreglass reinforced plastic, 1.0 m diameter × 0.6 m high and contained approximately 0.42 m^3^ water. Seawater was filtered (100 μm) and UV treated and pumped into the tanks at a mean flow rate of 3.5 l min^-1^ and oxygen saturation remained above 87%. The larvae hatched three to four days later, on 2 – 3 May. It has been demonstrated that cod embryos have an initial high mortality which becomes asymptotic at about eight days at 6.5°C, i.e. at 52 day degrees post fertilisation [27]. Hence, percentage survival at the applied sampling point (60 day degrees post fertilisation), is likely to represent hatching survival.

#### The fry stage

Initially, the larvae were fed rotifers (*Brachionus* spp.) enriched with Ori-Green (Skretting, Norway) and *Nannochloropsis sp*. (Reed Mariculture Inc., USA). Weaning from rotifers onto a micro particulate diet (Gemma Wean Diamond, Skretting, Norway) started at 37 dph and the larvae were co-fed rotifers for five days. At 94 dph, the cod fry were moved to three larger tanks. Three spawning tanks were used for this purpose. Though metamorphosis in cod is considered to take place when the fish are 12–15 mm [32], some aspects of metamorphosis require further time, such as the ability of the stomach to store and grind food particles, which developes gradually at 20 – 40 mm size [33]. Hence, the second sampling was conducted at 110 dph, when mean body lengths of fry were 5.08 cm (± 0.74 SD), 4.75 cm (± 0.82 SD) and 5.57 cm (± 0.82 SD) for replicate tanks 1, 2 and 3. Cannibalism was only observed on two occasions. Seawater temperature at sampling was 11.5°C. From each of the three replicate tanks, 300 fry were randomly netted, euthanised with metacaine (MS-222), preserved in 96% ethanol and stored at 4°C.

### Genotyping

Genomic DNA was extracted using the Real Pure Genomic DNA extraction kit (RBMEG02; Durviz). Three microsatellite loci, *Gmo*8, *Gmo*19 and *Tch*13 (Table 2), were co-amplified in 9 μl reactions containing 1 – 100 ng DNA template, 0.2 μM each forward and reverse primer, of which all forward primers were fluorescently labelled, 1 × Qiagen multiplex PCR buffer (contains a hotstart polymerase) and 0.5 × Qiagen Q-solution. Reaction conditions involved an initial denaturation step of 15 min at 95°C followed by 24 cycles of 30 s denaturation at 95°C, 3 min annealing at 60°C and 1 min extension at 72°C, and a final extension step of 30 min at 60°C. The amplified PCR products were processed on an ABI 3130*xL* Genetic Analyzer. Each well contained 2 μl PCR product (diluted 1:36), 7.90 μl Hi Di formamide solution and 0.10 μl GeneScan 500 LIZ® size standard (reagents from Applied Biosystems). GeneMapper version 4.0 (Applied Biosystems) was used to score the genotypes, which were all inspected visually. Positive and negative control samples were included in each plate and, to check consistency of results, 14% of the samples were re-amplified and re-run. Allele calls were consistent between runs for all markers.

**Table 2:** Primer and locus information for the three microsatellite markers used.

				Allele	No. Of	
	Repeat	Primer sequence		size	alleles in	
Locus	Motif	(5´→ 3´)	Dye	range (bp)	parental cod	Reference
*Gmo*8	GACA	F: GCA AAA CGA GAT GCA CAG ACA CC	NED	112–252	20	[34]
		R: TGG GGG AGG CAT CTG TCA TTC A				
*Gmo*19	GACA	F: CAC AGT GAA GTG AAC CCA CTG	VIC	122–206	15	[34]
		R: GTC TTG CCT GTA AGT CAG CTT G				
*Tch*13	GT	F: TTT CCG ATG AGG TCA TGG	6-FAM	78–158	18	[35]
		R: AAT CCA CTG GTG CAG ACC				

### Parentage analyses

Progeny were assigned to their respective parents by the exclusion-based parental assignment programme FAP [36]. Preliminary analysis employing the predictive function in FAP had confirmed that genotype data from the selected three highly variable microsatellite loci were of sufficient power (100% diagnostic) to assign parentage in all the reared groups. In practice, by applying a one allele mismatch tolerance, all except one of the 899 fry screened were unambiguously assigned to a single batch. The one remaining individual was assigned to a single batch by applying a two allele mismatch tolerance.

### Statistical analyses

Statistical analyses were conducted in Systat (version 11.0, Systat Software, Inc.; www.systat.com) and Microsoft Excel 2013. Data distributions were checked for conformance to normality using the Kolmogorov-Smirnov test. A multiple linear regression analysis was used to explore the relationships between survival rates of spawning batches through the embryo stage and two independent variables, egg diameter and female condition factor (*K*). To explore batch survival over the fry stage, χ^2^ goodness-of-fit tests [37] were used to compare observed versus expected numbers of progeny per female, calculated for each of the three replicate tanks. The Spearman Rank Correlation test was applied to evaluate the association between survival rates in the embryo and the post-metamorphosis fry stages.

## Results

### Differential batch survival at the embryo stage

Successful spawning / fertilisation was observed in all experimental tanks. The mean survival rate per batch was 69% (± 20% SD). Multiple linear regression analysis showed that egg diameter had a significant effect on embryo survival, accounting for 90% of the variation in embryo survival rates, while no significant effect of female condition factor on embryo survival was found (Table 3).

**Table 3:** Multiple linear regression analysis of the effect of egg diameter and female condition on survival in the embryo stage (*N* = 8), multiple *r*^2^ = 0.90.

	Coefficient	Standard Error	*t*	*P*
**Constant**	-379.24	75.03	-5.06	<0.01
**Egg diameter**	351.62	66.39	5.30	<0.01
**Female condition**	-2.18	11.46	-0.19	0.86

### Differential batch survival at the fry stage

**Fig 1:**
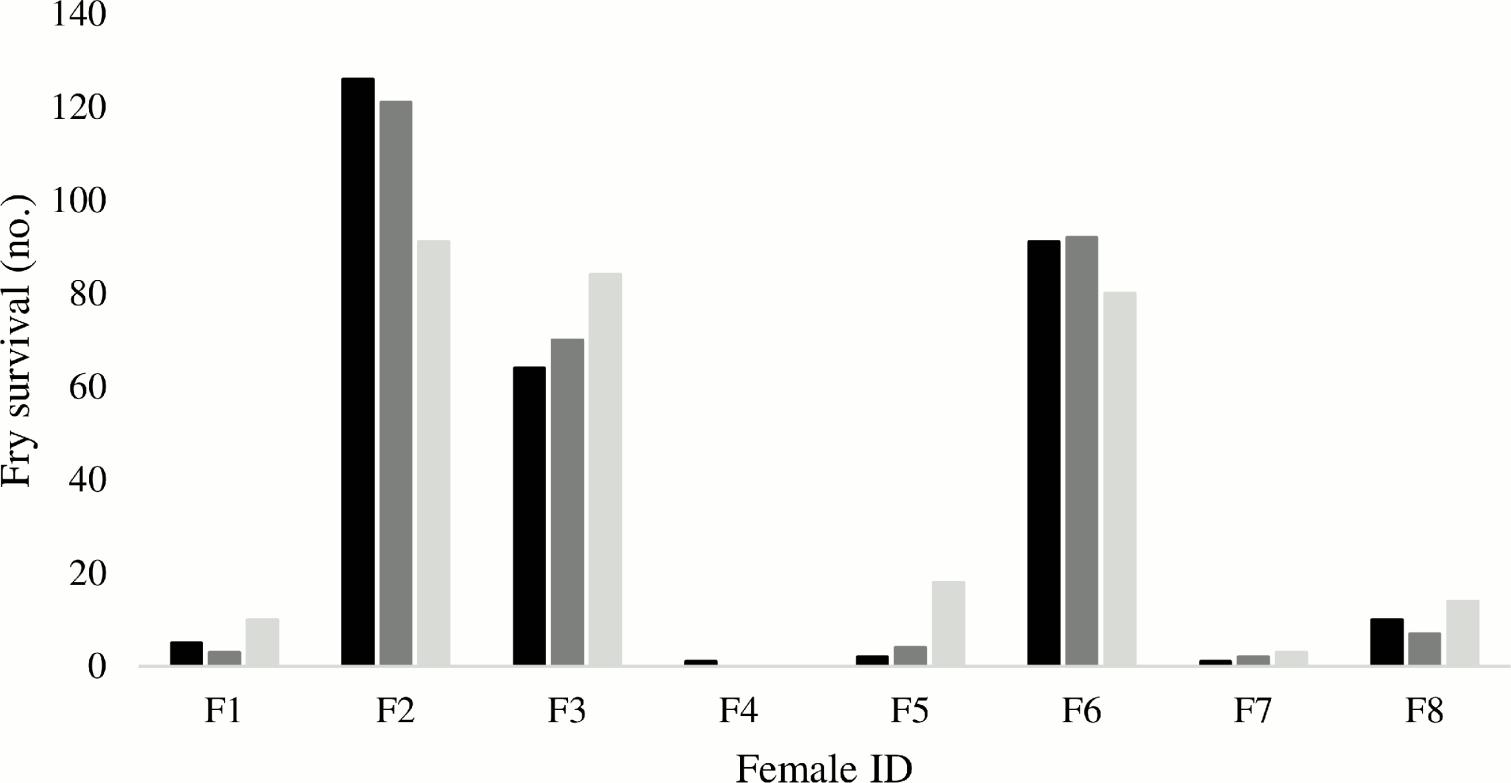
Survival numbers of post-metamorphosis progeny per replicate tank. Black, dark grey and light grey bars represent tank 1, 2 and 3, respectively.

Batch survival rates at 110 dph in each of the three replicate fry tanks, originally set up with equal contributions from each batch, were found to be highly skewed (Tank 1: χ^2^ = 456.9, *df* = 7, *P* < 0.0001; Tank 2: χ^2^ = 452.4, *df* = 7, *P* < 0.0001; Tank 3: χ^2^ = 296.4, *df* = 7, *P* < 0.0001). The pattern of batch survival was consistent over the three replicate tanks (Fig 1), with batches from three females (F2, F3 and F6) predominating; parenting a total of 91.1% of the progeny pooled over replicates. The residual 8.9% belonged to the five remaining females with the batch from female F4 only contributing to a single offspring of 899 progeny genotyped (Table 4). Hence, batches from the eight females formed two groups, one high (F2, F3 and F6; mean contribution 30% ± 7% SD of the fry), and one low survival group (F1, F4, F5, F7 and F8; mean contribution 2% ± 2% SD).

**Table 4:** Parental contribution to post-metamorphosis progeny, for the three replicate tanks (where applicable divided into contributions from each of two males; Table 1) and total over all three tanks.

Female	Replicate			Total per batch		
ID	1	2	3	No.	%	Cumulative (%)
F2	65/61	70/51	63/28	338	37.6	37.6
F6	91/0	92/0	80/0	263	29.3	66.9
F3	24/40	29/41	34/50	218	24.2	91.1
F8	10	7	14	31	3.4	94.5
F5	0/2	1/3	1/17	24	2.7	97.2
F1	2/3	3/0	5/5	18	2	99.2
F7	1	2	3	6	0.7	99.9
F4	0/1	0/0	0/0	1	0.1	100.0
**All**	300	299	300	899	100	100

Half-sib families were detected in four out of six possible batches (Tables 1 and 4), including two from the high survival group (F2 and F3). These two had been stocked with males of similar size and condition (Table 1). The remaining high survival batch (F6) contained only progeny from one of two possible males. While these did not differ significantly in body length, the contributing male had a higher condition factor (1.53 compared to 1.28 for the non-contributing male; Table 1). Again, observations were consistent over replicate tanks (Table 4).

**Fig 2:**
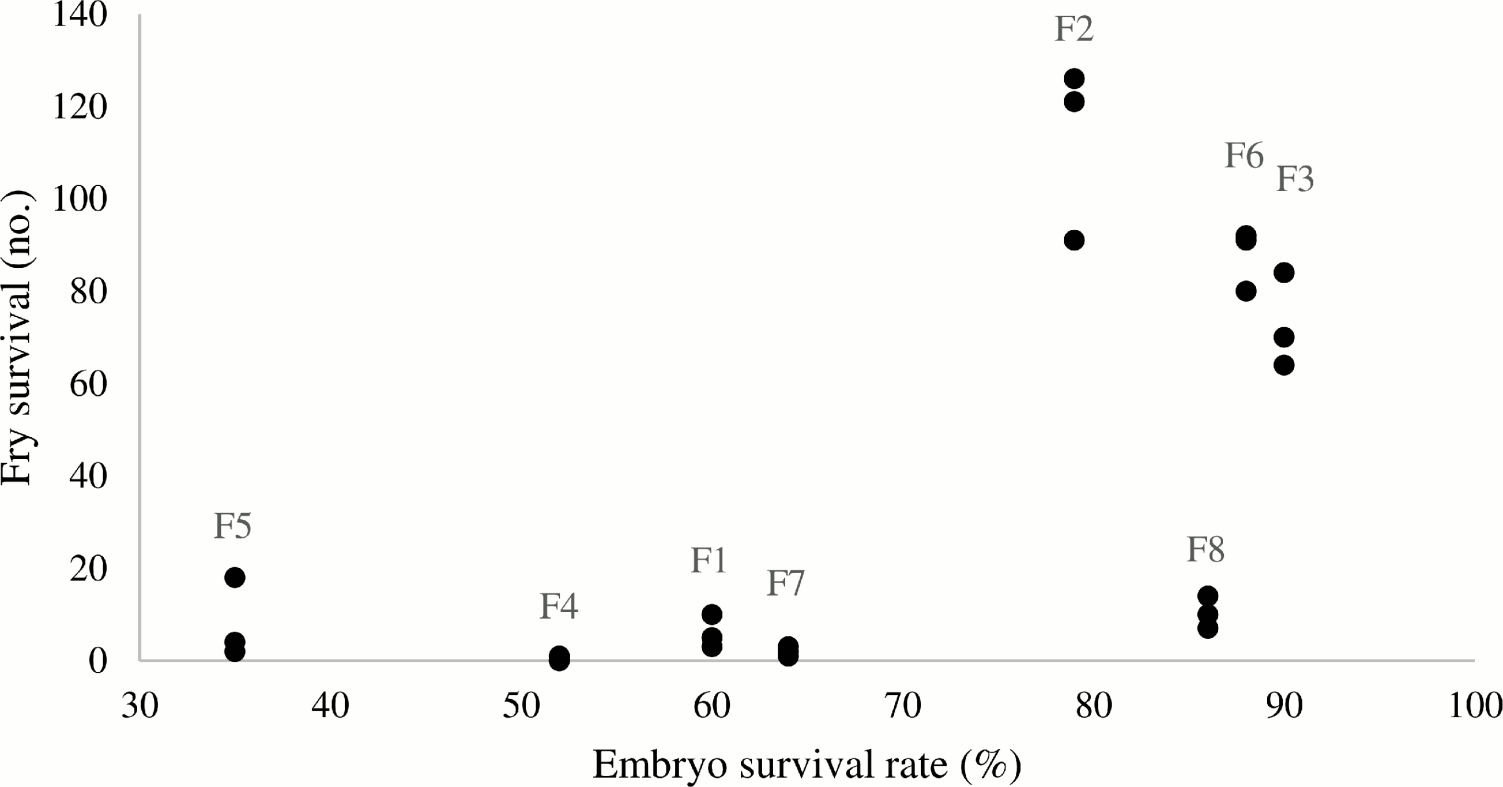
Scatterplot of survival in the fry stage versus survival in the embryo stage. For each observation of embryo survival, there are three replicate observations of fry survival. Labels above indicate batch identity.

A scatterplot of fry versus embryo survival indicated, with one exception (F8), that high survival in the embryo stage was associated with high survival in the fry stage and vice versa for low survival rates in the two stages (Fig 2). A Spearman Rank Correlation test showed a modest correlation between survival in the fry and embryo stages (*r*_s_ = 0.67; *P* < 0.10), although not significant at the 0.05 level.

## Discussion

Eight spawning batches from different females were investigated and the results show a large interbatch skew in survival of cod embryos and cod fry with a strong correlation between egg diameter and survival rates in the embryo stage. Female condition factor was not found to have a significant effect on embryo survival. Although not significant at the 0.05 level, the present data were too scarce to exclude a correlation between survival of embryos and survival of metamorphosed fry.

As pointed out by others [23], there are many potential egg quality markers, but for routine application it is important that such markers are both robust and practical to monitor / record. From this perspective, it is encouraging that 90% of the variation in egg survival rates could be predicted from egg diameter, an estimate that is easy to obtain. This observation is somewhat intriguing, as other studies, on cod and other species, did not find any association between egg size and hatching success [38]. However, in a study comparing mean egg size and hatching success among first- and second-time cod spawners, a significant effect of egg size on hatching success was found [39]. The lack of replicatation in the embryo stage as well as the lack to account for overall survival rates at the fry stage may introduce some uncertainty in our analyses. However, having replicate tanks for rearing to post-metamorphosis fry stage was a significant strength of the current study, since unidentified mortalities in the period from first feeding to metamorphosis appear to be the rule rather than the exception in intensive production of Atlantic cod [6]. Survival numbers of metamorphosed fry were consistent among all three replicate tanks, with batches from the same three females dominating. Thus, concerning fry survival, there were no adverse tank effects and the results truly demonstrated a strong batch effect.

While there have been several studies focusing on differential survival among cod families (for example [9,18,22]), to the authors´ knowledge, the current project is novel in that survival rates were evaluated for two different periods in development. Moreover, mixing progeny prior to hatch facilitated the process of transferring a precise number of individuals from each batch to the first-feeding tanks and, ultimately, allowed for more precise enumeration of progeny survival per batch. Though the present data were too scarce to conclude either way regarding a possible relationship between survival rates in the two developmental stages, an investigation of turbot (*Scophthalmus maximus*), another marine species, demonstrated that egg quality parameters (rates of fertilisation and of normal blastomeres) were predictive of viability and normal development up to the post-metamorphosis stage [40].

It is important to point out that the strong batch effect observed in the current study does not demonstrate an hereditary difference between families regarding progeny survival. In fact, whereas significant genetic variation has been demonstrated for body weight in Atlantic cod (heritability estimates between 0.3 and 0.5), heritability estimates concerning survival (in a setup involving 51 full-sib families) were found to be zero [41,42]. Having said that, such strong effects of batch survival may significantly alter family distributions in future generations and, hence, bear relevance to effective management of breeding populations.

Although many studies have documented a positive relationship between female size and / or condition and egg sizes of Atlantic cod [11,43,44], there is also strong batch effect on egg sizes, which generally decrease as the spawning season advances [21,43,45]. Furthermore, recruit spawners have been found to produce smaller eggs than repeat spawners [21]. In this study female condition factor was not significantly related to survival rate in the embryo stage. Similar results were also obtained in a previous study where no clear association was found between female pre-spawning condition and survival to first hatch [38]. Furthermore, these observations are in agreement with the biology of teleost fishes in general, where female age, but not female size, has been identified as a predictive factor of hatching success. However, in teleost fishes, female size is expected to affect the further viability of the progeny, through its effect on egg size [46]. Although results in cod are somewhat ambiguous [38], a relationship between female pre-spawning condition and embryo viability has been demonstrated [12]. Investigation of eggs from wild cod fertilised at sea [44] showed a significant effect of female condition on egg size as well as a significant positive relationship between egg sizes and larval sizes, early larval feeding and growth rates, but no significant association between egg sizes and growth rates at day 20. Similarly, for female condition, there was a significant positive relationship between female condition and early larval feeding, but not with larval growth rates at day 15 and day 20 [44]. Hence, the female effect appears to be most apparent in the early life stages and this may explain why the present study found no significant relationship between female condition and sizes and survival rates of metamorphosed cod fry. Alternative explanations for the lack of any significant relationships between female condition factors and larval viability indicators in the present study may lie in the fact that all females were in good condition, possibly making such effects more difficult to detect, and / or the fact that possible female effects were camouflaged by batch effects.

Whereas the focus of this paper is on aquaculture production of cod, the results presented may also be important from a conservation perspective. Despite the wide distribution and great dispersal abilities of marine fish, effective population sizes are generally between two and six orders of magnitude smaller than census population sizes. For Atlantic cod, estimated effective population sizes range from *c*. 100 to *c*. 2000 individuals, numbers that are sufficiently small to put the populations at risk of losing genetic variability through genetic drift (see [47] and references therein). The most likely reason for the relatively small effective population sizes is a skewed reproductive success among spawners, that is usually seen as a demographic process [47]. However, the current study demonstrates a strong batch component concerning early survival and adds to a few studies suggesting that genotype-specific survival could contribute to the relatively small effective population sizes seen for many marine fish [9,18,19,20,47,48]. The effective population size has not been estimated for the current study population, the Faroe Bank cod, but the census population size is historically low at the moment and there is almost a total moratorium on commercial fishing of this population [49].

In conclusion, the data demonstrated a strong batch component concerning survival rates of embryos and metamorphosed fry and egg size was found to be a useful indicator of survival rates in the embryo stage. As the data were too scarce to exclude either way regarding a possible association between survival rates in the two stages, a possible correlation between egg size and later viability could not be excluded.

## References

[1] Rosendal GK, Olesen I, Tvedt MW. Evolving legal regimes, market structures and biology affecting access to and protection of aquaculture genetic resources. Aquaculture 2013;402-403:97–105. 10.1016/j.aquaculture.2013.03.026

[2] FAO. Fishery Statistical Collections. Rome. http://www.fao.org/fishery/statistics/global-aquaculture-production/en. Accessed on 5 May 2016.

[3] Karlsen Ø, Norberg B, Kjesbu OS, Taranger GL. Effects of photoperiod and exercise on growth, liver size, and age at puberty in farmed Atlantic cod (*Gadus morhua* L.). ICES J Mar Sci. 2006;63:355–364. 10.1016/j.icesjms.2005.10.013

[4] Rosenlund G, Skretting M. Worldwide status and perspective on gadoid culture. ICES J Mar Sci. 2006;63:194–197. 10.1016/j.icesjms.2005.11.012

[5] Gunnarsli KS, Toften H, Mortensen A. Effects of nitrogen gas supersaturation on growth and survival in larval cod (*Gadus morhua* L.). Aquaculture. 2009;288:344–348. 10.1016/j.aquaculture.2008.06.029

[6] Kjesbu OS, Taranger GL, Trippel EA. Gadoid mariculture: development and future challenges. ICES J Mar Sci. 2006;63:187–191. 10.1016/j.icesjms.2005.12.003

[7] Teletchea F, Fontaine P. Levels of domestication in fish: implications for the sustainable future of aquaculture. Fish Fish. 2014;15:181–195. 10.1111/faf.12006

[8] Halldórsson JE, Björnsson B, Gunnlaugsson SB. Feasibility of ranching coastal cod (*Gadus morhua*) compared with on-growing, full-cycle farming and fishing. Mar Pol. 2012;36(1):11–17. 10.1016/j.marpol.2011.03.001

[9] Hutchings JA, Bishop TD, McGregor-Shaw CR. Spawning behaviour of Atlantic cod (*Gadus morhua*: evidence of mate competition and mate choice in a broadcast spawner. Can J Fish Aquat Sci. 1999;56:97–104.

[10] Nordeide JT, Folstad I. Is cod lekking or a premiscuous spawner? Fish Fish 2000;1:90–93. 10.1046/j.1467-2979.2000.00005.x

[11] Kjesbu OS. The spawning activity of cod, *Gadus morhua* L. J Fish Biol. 1989;34:195–206.

[12] Kjesbu OS, Klungsoyr J, Kryvi H, Witthames PR, Greer Walker M. Fecundity, atresia, and egg size of captive Atlantic cod (*Gadus morhua*) in relation to proximate body composition. Can J Fish Aquat Sci. 1991;48:2333–2343.

[13] Bekkevold D, Hansen MM, Loeschcke V. Male reproductive competition in spawning aggregations of cod (*Gadus morhua*, L.). Mol Ecol. 2002;11:91–102. 10.1046/j.0962-1083.2001.01424.x 11903907

[14] Trippel EA. Estimation of male reproductive success of marine fishes. J Northwest Atl Fish Sci. 2003;33:81–113.

[15] Rakitin A, Ferguson MM, Trippel EA. Male reproductive succes and body size in Atlantic cod *Gadus morhus* L. Mar Biol. 2001;138:1077–1085. 10.1007/s002270100551

[16] Armitage D, Maguire J, Treasurer J, Cross TF. Parental assignment in farmed cod (*Gadus morhua*) progeny in mass spawning using microsatellite DNA loci reveals major disparity in family representation [abstract]. Aquaculture. 2007;272 Suppl 1:S242 10.1016/j.aquaculture.2007.07.026

[17] Rowe S, Hutchings JA, Skjæraasen JE. Nonrandom mating in a broadcast spawner: mate size influences reproductive success in Atlantic cod (*Gadus morhua*). Can J Fish Aquat Sci. 2007;64:219–226. 10.1139/F06-182

[18] Herlin M, Delghandi M, Wesmajervi M, Taggart JB, McAndrew BJ, Penman DJ. Analysis of the parental contribution to a group of fry from a single day of spawning from a commercial Atlantic cod (*Gadus morhua*) breeding tank. Aquaculture. 2008;274:218–224. 10.1016/j.aquaculture.2007.11.034

[19] Wesmajervi MS, Westgaard J-I, Delghandi M. Evaluation of a novel pentaplex microsatellite marker system for paternity studies in Atlantic cod (*Gadus morhua*). Aquac Res. 2006;37:1195–1201. 10.1111/j.1365-2109.2006.01549.x

[20] Rudolfsen G, Figenschou L, Folstad I, Nordeide JT, Søreng E. Potential fitness benefits from mate selection in the Atlantic cod (*Gadus morhua*). J Evol Biol. 2005;18(1),172–179. 10.1111/j.1420-9101.2004.00778.x 15669974

[21] Kjesbu OS, Solemdal P, Bratland, Fonn M. Variation in annual egg production in individual captive Atlantic cod (*Gadus morhua*). Can J Fish Aquat Sci. 1996;53:610–620.

[22] Hansen LA, Damsgård B, Delghandi M. Spawning behaviour and reproductive success in cod, *Gadus morhua* L. [abstract]. Gadoid Mariculture: Development and Future Challenges Conference; 2004 June 13-16; Bergen, Norway. Copenhagen: ICES.

[23] Hansen ØJ, Puvanendran V. Fertilization success and blastomere morphology as predictors of egg and juvenile quality for domesticated Atlantic cod, *Gadus morhua*, broodstock. Aquac Res. 2010;41:1791–1798. 10.1111/j.1365-2109.2010.02506.x

[24] Pickova J, Dutta PC, Larsson P-O, Kiessling A. Early embryonic cleavage pattern, hatching success, and egg-lipid fatty acid composition: comparison between two cod (*Gadus morhua*) stocks. Can J Fish Aquat Sci. 1997;54:2410–2416.

[25] Nocillado JN, Peñaflorida VD, Borlongan IG. Measures of egg quality in induced spawns of the Asian sea bass, *Lates calcarifer* Bloch. Fish Physiol Biochem. 2000;22:1–9.

[26] Bobe J, Labbé C. Egg and sperm quality in fish. Gen Comp Endocr. 2010;165(3):535–548. 10.1016/j.yg.cen.2009.02.011 19272390

[27] Avery TS, Killen SS, Hollinger TR. The relationship of embryonic development, mortality, hatching success, and larval quality to normal or abnormal early embryonic cleavage in Atlantic cod, *Gadus morhua*. Aquaculture. 2009;289:265–273. 10.1016/j.aquaculture.2008.12.011

[28] Støttrup JG. (2002). Torskeopdræt – forskningsresultater og kundskab om torskeopdræt. Charlottenlund, Denmark: Danish Institute for Fisheries Research; 2002 DFU Report No.: 107– 02.

[29] Otterå H, Taranger GL, Borthen J. Oppdrett av Torsk Bergen, Norway: Norsk Fiskeoppdrett AS; 2005.

[30] Steingrund P, Hansen B, Gaard E. Cod in Faroese waters. In: Brander K, editor. Spawning and Life History Information for North Atlantic Cod Stocks. ICES Cooperative Research Report 274. Copenhagen, Denmark: ICES; 2005. pp. 50–55.

[31] Salvesen I, Vadstein O. Surface disinfection of eggs from marine fish: evaluation of four chemicals. Aquacult Int. 1995;3(3):155–171. 10.1007/BF00118098

[32] Brander K. Spawning and Life History Information For North Atlantic Cod Stocks. ICES Cooperative Research Report 274. Copenhagen, Denmark: ICES; 2005. pp. 1–152.

[33] Pedersen T, Falk-Petersen IB. Morphological changes during metamorphosis in cod (*Gadus morhua* L.) with particular reference to the development of the stomach and pyloric caeca. J Fish Biol. 1992;41(3):449–461. 10.1111/j.1095-8649.1992.tb02673.x

[34] Miller KM, Le KD, Beacham TD. Development of tri- and tetranucleotide repeat microsatellite loci in Atlantic cod (*Gadus morhua*). Mol Ecol. 2000;9(2):238–239. 10.1046/j.1365-294x.2000.00804-2.x 10672170

[35] O’Reilly PT, Canino MF, Bailey KM, Bentzen P. Isolation of twenty low stutter di- and tetranucleotide microsatellites for population analyses of walleye pollock and other gadoids. J Fish Biol. 2000;56:1074–1086. 10.1006/jfbi.2000.1230

[36] Taggart JB. FAP: an exclusion-based parental assignment with enhanced predictive functions. Mol Ecol Notes 2007;7:412–415. 10.1111/j.1471-8286.2006.01616.x

[37] Walpole RE, Myers RH, Myers SL, Ye K. Probability & Statistics for Engineers & Scientists 9th ed. Boston, USA: Prentice Hall; 2012.

[38] Ouellet P, Lambert Y, Bérubé I. Cod egg characteristics and viability in relation to low temperature and maternal nutritional condition. ICES J Mar Sci. 2001;58:672–686. 10.1006/jmsc.2001.1065

[39] Trippel EA. Egg size and viability and seasonal offspring production of young Atlantic cod. Trans Am Fish Soc. 1998;127(3):339–359. 10.1577/1548-8659(1998)127<0339:ESAVAS>2.0.CO;2

[40] Kjørsvik E, Hoehne-Reitan K, Reitan KI. Egg and larval quality criteria as predictive measures for juvenile production in turbot (*Scophthalmus maximus* L.). Aquaculture 2003; 227:9–20. 10.1016/S0044-8486(03)00492-7

[41] Gjerde B, Terjesen BF, Barr Y, Lein I, Thorland I. Genetic variation for juvenile growth and survival in Atlantic cod (*Gadus morhua*). Aquaculture 2004;236:167–177. 10.1016/j.aquaculture.2004.03.004

[42] Kristjánsson T, Arnason T. Review Article. Heritability of economically important traits in the Atlantic cod *Gadus morhua* L. Aquac Res 2016;47:349–356. 10.1111/are.12496

[43] Chambers RC, Waiwood KG. Maternal and seasonal differences in egg sizes and spawning characteristics of captive Atlantic cod, *Gadus morhua*. Can J Fish Aquat Sci 1996;53:1896–2003.

[44] Marteinsdóttir G, Steinarsson A. Maternal influence on the size and viability of Iceland cod *Gadus morhua* eggs and larvae. J Fish Biol. 1998;52,1241–1258. 10.1111/j.1095-8649.1998.tb00969.x

[45] Otterå H, Agnalt A-L, Jørstad KE. Differences in spawning time of Atlantic cod from four regions of Norway, kept under identical conditions. ICES J Mar Sci 2006;63:216–223. 10.1016/j.icesjms.2005.11.004

[46] Kamler E. Parent–egg–progeny relationships in teleost fishes: an energetics perspective. Rev Fish Biol Fish. 2005;15:399–421. 10.1007/s11160-006-0002-y

[47] Hauser L, Carvalho GR. Paradigm shifts in marine fisheries genetics: ugly hypotheses slain by beautiful facts. Fish Fish. 2008;9:333–362. 10.1111/j.1467-2979.2008.00299.x

[48] Planes S, Romans P. Evidence of genetic selection for growth in new recruits of a marine fish. Mol Ecol. 2004;13(7):2049–2060. 10.1111/j.1365-294X.2004.02202.x 15189225

[49] ICES. Report of the North-Western Working Group (NWWG). ICES CM 2014/ACOM 2014;07:1–915.

